# Crop genomic selection with deep learning and environmental data: A survey

**DOI:** 10.3389/frai.2022.1040295

**Published:** 2023-01-10

**Authors:** Sheikh Jubair, Mike Domaratzki

**Affiliations:** ^1^Department of Computer Science, University of Manitoba, Winnipeg, MB, Canada; ^2^Department of Computer Science, University of Western Ontario, London, ON, Canada

**Keywords:** genomic selection, machine learning, GxE, environment, MET, deep learning

## Abstract

Machine learning techniques for crop genomic selections, especially for single-environment plants, are well-developed. These machine learning models, which use dense genome-wide markers to predict phenotype, routinely perform well on single-environment datasets, especially for complex traits affected by multiple markers. On the other hand, machine learning models for predicting crop phenotype, especially deep learning models, using datasets that span different environmental conditions, have only recently emerged. Models that can accept heterogeneous data sources, such as temperature, soil conditions and precipitation, are natural choices for modeling GxE in multi-environment prediction. Here, we review emerging deep learning techniques that incorporate environmental data directly into genomic selection models.

## 1. Introduction

Production of sufficient food for the increasing world population is a major concern. Industrialization and development of infrastructure in developing countries are causing a shortage of land for growing populations in urban areas, which leads to unplanned expansion of cities into agricultural land (Azadi et al., 2011). Soil erosion due to water, wind, or excessive use for cultivation affects the topsoil and fertility, thus reduces crop production. A large amount of surface and groundwater has already been used, causing a decrease in groundwater level (Van Meijl et al., 2018). Global temperature is increasing and heat waves have become more frequent, which leads a significant decrease in crop production (Bourgault et al., 2018; Nawaz and Chung, 2020). Though several regions will benefit from the effect of climate change, especially because of the increase in temperature, overall food production will decrease by 2050 (Van Meijl et al., 2018).

These problems will increase the price of the food and people, especially in developing countries, will suffer from hunger and deficiency in nutrition, causing low growth in children or low weight (Linehan et al., 2012; United Nations, 2019; Nawaz and Chung, 2020). It is projected by the UN that by 2050, the world population will reach 9.7 billion and to accommodate this vast number of people, a large amount of new agricultural land will be needed (Searchinger et al., 2019). This will lead to the “more people, less agricultural land” problem (United Nations, 2019; Nawaz and Chung, 2020). To ensure food security and keep the food affordable to everyone, by 2050, we will need to increase our food production by 50% of our current production (Nawaz and Chung, 2020).

To face the challenge of food production in the future, selection of varieties with desired phenotypes from a collection of varieties of a crop is essential to breeders, as the right selection can lead to improvements such as drought resistance, biotic and abiotic stress resistance, yield improvement and disease resistance (Varshney et al., 2017). While the amount of water, fertilizer, pest control, and sound production practices contribute to the environment for the plant, the genotype of the plant defines the ability to produce a desired phenotypic value within that environment (Milton, 1979). Thus, as environmental factors and breeding practices are standardized and measured, it is vital to create improved varieties for that environment.

Genomic selection (GS), first defined by Meuwissen et al. (2001), is a marker-assisted selection method that uses dense whole-genome molecular markers to improve the quantitative traits of an organism such as a crop or livestock by identifying the top germplasms. That is, GS is a computational tool for choosing the most advantageous individuals from a set of varieties and has the potential to save money and time by accelerating improvements to crops or livestock (Acquaah, 2009; Varshney et al., 2017).

GS for single environment trials employs GS to identify top individuals to create a new variety for a specific environment (Meuwissen et al., 2001; Heffner et al., 2009; Crossa et al., 2017; Jubair et al., 2021). If the environment changes, single environment GS does not guarantee that the new variety will have the desired outcome in that new environment (Oakey et al., 2016). GS for multi-environment trial is a generalization that is able to identify top organisms even if the environment is new (Washburn et al., 2021). In this survey, we focus on applications of deep learning in both single and multi-environment trial and analyze the differences between single environment and multi-environment models. In particular, we are interested in those multi-environment models that incorporate data such as hourly temperature, rainfall or other time series data from environments into deep learning models to improve prediction. The reader may wish to consult existing reviews of genomic selections for material focused on statistical models of single environment (Wang et al., 2018; van Dijk et al., 2021; Anilkumar et al., 2022) and multi-environment trials (Tong and Nikoloski, 2021; van Dijk et al., 2021). Additionally, several reviews cover fully the use of machine learning models for single environment trials (Montesinos-López et al., 2021; Tong and Nikoloski, 2021; van Dijk et al., 2021; Anilkumar et al., 2022; Danilevicz et al., 2022). Xu et al. (2022) also review GS and describe the potential for the use of multiple sources of data beyond genomic data, including enviromental data. This includes the use of machine learning models. In contrast, multi-environment deep learning approaches are an emerging area that enable detailed weather data to be incorporated directly into the model (Khaki and Wang, 2019; Khaki et al., 2020; Lin et al., 2020; Shook et al., 2020). Our survey focuses specifically on recent works involving this latter class of models that employs genomic and weather data together to inform deep learning models and predict phenotypes.

Traditionally, we can identify two broad approaches to GS. Linear methods such as BLUP and variants (Burgueño et al., 2012; Bandeira e Sousa et al., 2017; Cuevas et al., 2017, 2019; Ferrão et al., 2017; Howard et al., 2019; Millet et al., 2019) explicitly model the phenotype in terms of contributions from different factors, including pedigree, individual markers or distinct site-years. Typically, these models perform well for additive traits due to the linear nature of the models. On the other hand, machine learning models, such as Random Forests (RFs) (Holliday et al., 2012; Ali et al., 2020; Sawitri et al., 2020), Support Vector Machines (SVMs) (Ogutu et al., 2011; Wang et al., 2019) and Neural Networks (NNs) (Jubair and Domaratzki, 2019; Pérez-Enciso and Zingaretti, 2019) can model traits in non-linear but typically opaque ways. For a complete introduction to machine learning and deep learning (DL), see Emmert-Streib et al. (2020) or Dey (2016). In this paper, our focus is on the deep learning methods in this area.

Crops respond differently in different environmental conditions (Millet et al., 2019), an effect known as genome by environment interaction (GxE). This leads to differences in production quantity or quality (Cuevas et al., 2017). In a single environment trial, it is typically assumed that the environment is constant, thus, there is no effect of environment on genotypes. A number of deep learning methods for single environment trials have been published (McDowell, 2016; Rachmatia et al., 2017; Ma et al., 2018; Jubair and Domaratzki, 2019; Zingaretti et al., 2020; Jubair et al., 2021; Montesinos-Lopez et al., 2021). These methods differ in their deep learning architectures and focus on how they capture the genetic information. Multi-environment models can be thought of an extension of single environment trial as the models consider the interaction between environment and genome. Though multi-environment trials are an extension of single-environment GS, there are very few deep learning methods that have been developed for this problem (Montesinos-López et al., 2018, 2019b; Khaki and Wang, 2019; Khaki et al., 2020; Lin et al., 2020; Shook et al., 2020) that take GxE interaction in crops into account because of the complexity in incorporating the environmental interaction into the model and lack of complete environmental data. In the past 3 years, new research has demonstrated the potential of incorporating environmental information into deep learning models for GS (Khaki and Wang, 2019; Khaki et al., 2020; Lin et al., 2020; Shook et al., 2020). This survey focuses specifically on deep learning methods for integrating weather data into GS. The ability to integrate heterogenous data into a model is a known strength of machine learning models in general, and deep learning models in particular. However, this research is one facet of a large, active research community that seeks to improve GS accuracy, using various models, through integration of types of environmental data (Costa-Neto et al., 2022; Montesinos-López et al., 2022; Putra et al., 2022; Song et al., 2022).

In this survey, our aim is to provide a comprehensive overview of genomic selection process with deep learning that starts from data and ends with creating a new variety for both single and multi-environment trial. To do this, (i) we provide an overview of different data of GS and how these data need to be processed, (ii) discuss popular components of deep learning models typically employed in GS and then (iii) review existing deep learning architectures and motivation behind them for both single and multi-environment trials.

## 2. Datasets for GS

Crop organisms are usually genotyped using high throughput sequencing technology that uses a large number of genomic markers to cover the whole genome of that organism (Goddard and Hayes, 2007; Heffner et al., 2009; Crossa et al., 2017). These markers are usually represented by categorical values based on their zygosity or sequencing technology. For example, a diploid organism is usually represented by 1, 0 and −1 where 1 and −1 represent homozygous allele and 0 represents heterozygous allele. If DArT assays are used for sequencing, SNPs are represented by binary values, indicating a gene's presence or absence (Crossa et al., 2016b; Jubair and Domaratzki, 2019). Table 1 shows an example dataset.

**Table 1:** An example of genotyped data.

**Genotype**	***M*** **_1_**	***M*** **_2_**	**…**	**…**	***M*** **_*D*_**
*Geno*_1_	1	–1			–1
*Geno*_2_	0	1			1
**…**	0	-1	…	…	1
**…**	0	0	…	…	1
*Geno*_*N*_	–1	0	…	…	0

In the column header, *M* means markers. This dataset contains *D* markers and *N* genotypes. Thus, each line is represented by *D* markers. Each of these markers can have one of the three values: 1, 0, and –1.

As the data may contain uninformative markers and missing values, the genotyped data often need pre-processing. The preprocessing steps may involve removing uninformative markers, imputation of missing values and representing the features in some other forms. If the minor allele frequency ≤ 5% (Ma et al., 2018; Jubair et al., 2021) or more than 30% values are missing, then the marker is usually removed as those markers do not bear any relevant information. To replace the missing values, one popular imputation techniques is k-nearest neighbor. For example, at first, the k-nearest genotypes of the genotype of interest are identified. From those genotypes, the missing value is replaced by the most frequent value for the specific marker.

Most neural networks consist of a linear equation that multiplies a weight vector with a feature vector (LeCun et al., 2015; Dong et al., 2021). If a feature is represented with a zero, it means the feature will not have any influence on final outcome as the resulting multiplication between the weight and feature will also be zero. Thus, providing traditional marker data as input to the deep learning models may result in a loss of information. This may lead us to think that representing the allele with other categorical values such as 1, 2, and 3 will solve this issue. This leads to another problem as multiplying weights with a high value of a specific allele may mislead the deep learning model to give higher priority to that specific allele. To solve these problems, one-hot encoded vector (Liu et al., 2019b) or Hardy-Weinberg equlibrium can be used to represent markers (Jubair et al., 2021). A one-hot encoded vector is an *n* dimensional sparse vector where *n* is the number of alleles of a specific marker. Each allele of a marker is associated with a specific position in the vector. If that allele is present in the marker, the specific position for the allele is represented with 1 and other positions with 0. Sometimes, an extra position is also added to the one hot encoded vector to represent missing values (Liu et al., 2019b). As an alternative to categorical encoding and one hot encoded representation, markers can also represented by their allele frequency (Jubair et al., 2021), which can be obtained following the Hardy-Weinberg equilibrium formula. For example, suppose, in 10 genotypes, allele *AA*, *Aa*, and *aa* for a specific marker occurs 6, 3, and 1 times, respectively. Then the frequency of *AA*, *Aa*, and *aa* is 0.6, 0.3, and 0.1, respectively.

The environment of crops comprises weather, soil and field management data. Weather information, such as maximum and minimum temperature, precipitation, vapor pressure, wind speed and radiation, plays an essential part in GS for multi-environmental trials (Khaki and Wang, 2019; Gangopadhyay et al., 2020; Khaki et al., 2020; Shook et al., 2020). Weather information can be integrated as daily, weekly, monthly or yearly averages based on the architecture of the deep learning model (Khaki and Wang, 2019; Khaki et al., 2020; Washburn et al., 2021). In addition, soil information such as percentage of clay, silt and sand, water capacity, soil pH, number of irrigation, organic matter, and cation-exchange capacity also plays a vital role (Washburn et al., 2021). Sometimes, field management information such as the number of irrigations, sowing pattern of crops, amount of water used in irrigation, and amount of fertilizer or insecticide applied is also recorded. These can also be integrated with soil data as they carry valuable information (Washburn et al., 2021). As the variables from environmental information are in different ranges, these variables are usually scaled by zero-centering as a pre-processing step. Table 2 shows an example of genotyped and environmental data after pre-processing.

**Table 2:** An example of genotyped and environmental data after pre-processing in a tabular format.

		**Markers**			**Weather variables**			**Soil variables**			**Field management**		
**Envs**	**Geno**	***M***_**1**_	**…**	***M***_***D***_	***W***_**1**_ ***t*** **= 1**	**…**	***W***_***w***_ ***t*** **=** ***T***	***S***_**1**_	**…**	***S***_***s***_	***F***_**1**_	**…**	***F***_***f***_
*Env*_1_	*Geno*_1_	0.6	…	0.4	0.32	…	0.27	0.2	…	0.15	0.4	…	0.6
*Env*_1_	*Geno*_2_	0.2	…	0.4	0.32	…	0.27	0.2	…	0.15	0.2	…	0.21
*Env*_2_	*Geno*_3_	0.6	…	0.4	0	…	0.4	0.32	…	0.24	0.25	…	0.05
*Env*_3_	*Geno*_4_	0.6	…	0.2	0.65	…	0.1	0.3	…	0.31	0.4	…	0.1
**…**	**…**	…	…	…	…	…	…	…	…	…	…	…	…
**…**	**…**	…	…	…	…	…	…	…	…	…	…	…	…
***Env*** **_*k*_**	***Geno*** **_*n*_**	0.2	…	0.4	0.65	…	0.1	0.3	…	0.31	0.2	…	0.1

In this example, each genotype has *D* markers after removing minor alleles and imputing missing values. Marker values are represented by their allele frequency. There are *w* weather variables where each weather variables are divided in *T* time steps. Apart from the weather variables, there are *s* soil variables and *f* field management variables too. All the data are normalized.

## 3. Deep learning

in recent years, Deep Learning has emerged as a leading paradigm for supervised machine learning tasks. Significant innovation has occurred in diverse areas like Natural Language Processing, Computer Vision, and Bioinformatics (LeCun et al., 2015; Li et al., 2020; Dong et al., 2021). The dominant paradigm in DL is a network. A deep learning network is made up of blocks and each block has several different types of layers. A block usually contains multiple layers of one or more neural networks, activation function, normalization layer and regularization layer (LeCun et al., 2015; Dong et al., 2021). In this section, we discuss each of the layers of neural network blocks and describe the function of the most common layers. It is worth mentioning that we chose these layers based on their usage in previous research conducted in GS.

**Figure 1:**
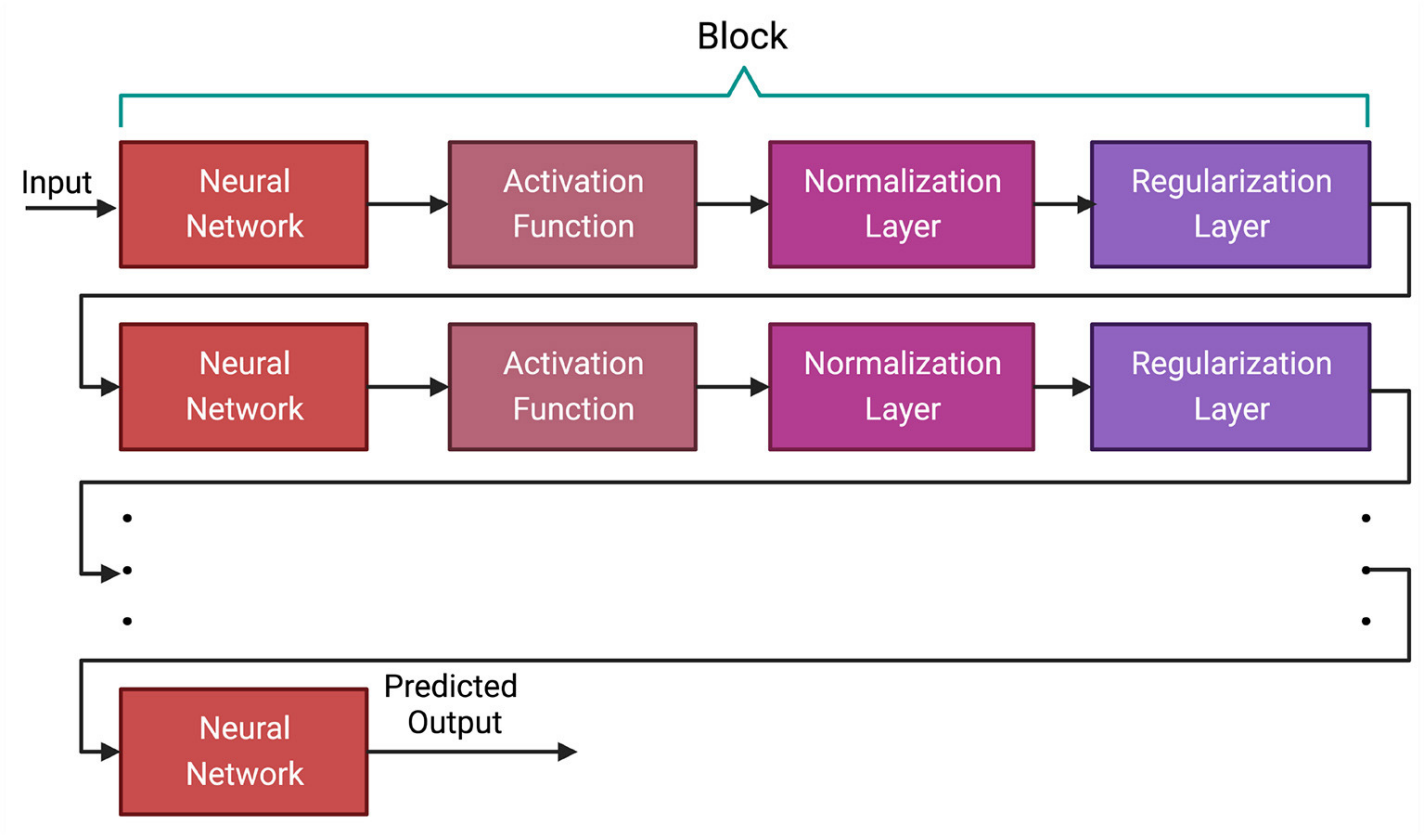
General architecture of a deep learning algorithm. All the layers between the first and last layer are called hidden layers. The first layer is the input layer and the last layer is the output layer. A neural network layer is typically followed by an activation function and then by normalization and regularization layers. Based on the architecture of the deep learning model, some of these layers, such as normalization and regularization layers, may not be present in a block.

In a deep learning model, the layers between the input and output are called hidden layers. Each layer consists of several nodes called neurons where we receive input and perform computation on the data from previous layers. Typically, the neural network layer contains one or more feed-forward (Bebis and Georgiopoulos, 1994), convolution (Kim, 2017; Kiranyaz et al., 2021) or Long Short-Term Memory (LSTM) (Hochreiter and Schmidhuber, 1997; Yu et al., 2019b) layers (discussed in Section 3.1). As these neural networks are generally linear functions, activation functions such as ReLU and sigmoid are applied to the output of the neural network layer to introduce non-linearity (discussed in Section 3.2). Normalization and regularization layers such as L1, L2 and dropout are applied after the activation layer to generalize the model to avoid overfitting (discussed in Section 3.3). Figure 1 shows the general architecture of a deep learning method.

### 3.1. Neural networks

#### 3.1.1. Fully connected neural networks

**Figure 2:**
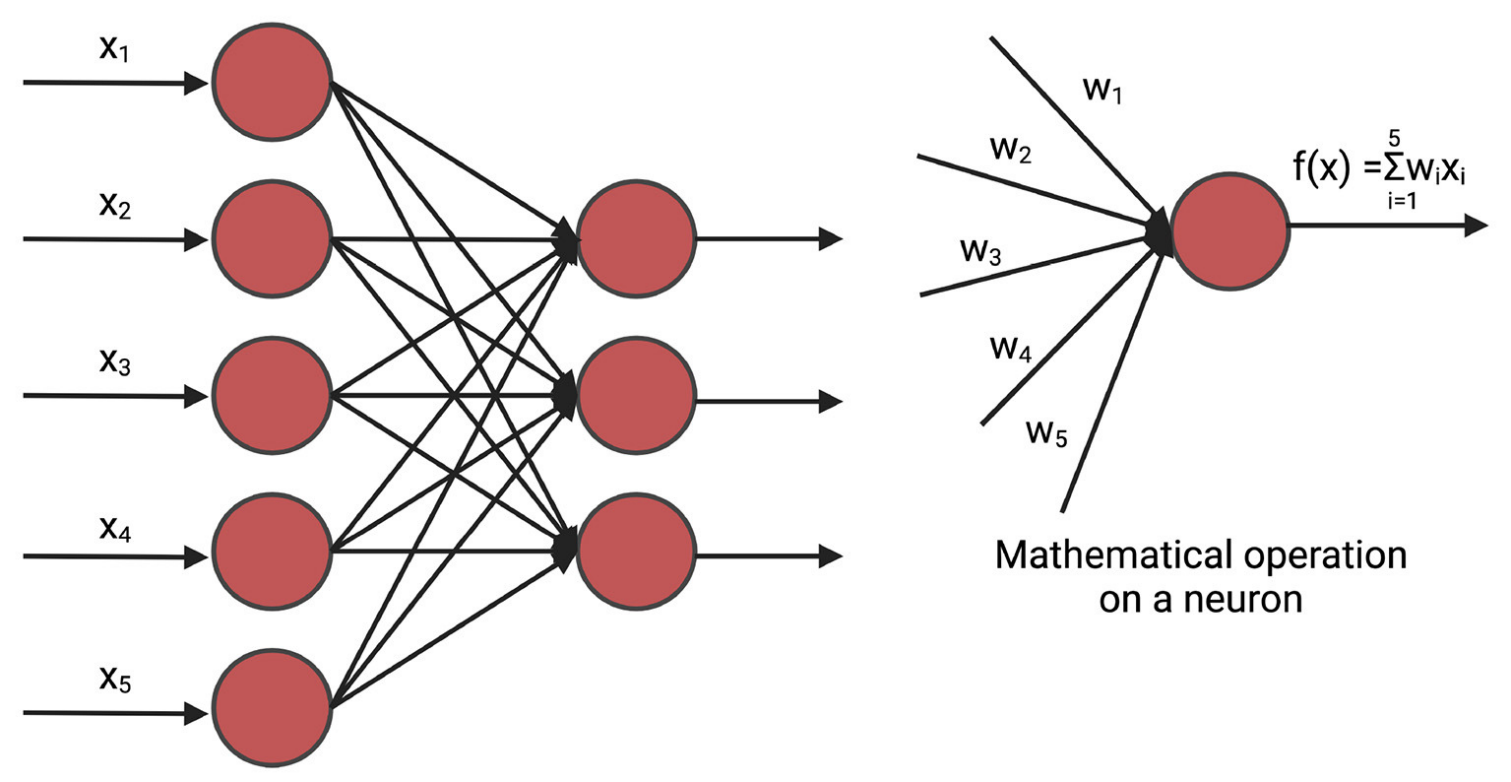
An example of fully connected layer. *x*_*i*_ is the input and *w*_*i*_ is the weight. The weights are initialized randomly and are optimized iteratively for prediction. In GS, *x*_*i*_ is the marker.

A fully connected neural network (FNN), often referred to as a linear layer, is an Artificial Neural Network where all the neurons of the previous layer are connected to each neuron of the current layer. The mathematical operation of the fully connected neural network can be compared to *n* linear regression methods (Montgomery et al., 2021) where *n* is the number of hidden neurons of the current layer. A deep fully connected neural network is often called Multi-Layer Perceptron (MLP). Figure 2 shows a fully connected network.

MLPs have been applied to predict phenotypes both in single environment trial (Gianola et al., 2011; González-Camacho et al., 2016; Jubair and Domaratzki, 2019; Montesinos-López et al., 2019a; Jubair et al., 2021) and multi-environment trial Montesinos-López et al. (2018), Khaki and Wang (2019). In case of single environment trials, the input is the genotyped data of crops. When the prediction of phenotypes is for multi-environment trials, additional information such as environmental data are concatenated with the genotyped data. This concatenated vector is the input of the feed-forward networks and the output is the environment-specific predicted yield (Khaki and Wang, 2019).

#### 3.1.2. Convolutional neural networks

Convolutional neural networks are a successful model of DL that employ convolution operations to incorporate targeted regions of input in decision making (Li et al., 2021). A convolution operation summarizes point-wise multiplication between a small kernel that slides over the input of the convolution layer. The weights of the kernels are shared across all the sliding windows. These kinds of neural networks are known for capturing local information within the data since, in each sliding window, the network is on a small subset of the data (LeCun et al., 2015; Dong et al., 2021). Convolution operations were first developed in vision to help identify features of an image in a restricted window as the spatial information in the image plays a vital role in most vision applications (Dong et al., 2021; Li et al., 2021). The applications of convolutional neural networks have also been extended to other domains such as GS (Ma et al., 2018; Jubair and Domaratzki, 2019; Liu et al., 2019b; Zingaretti et al., 2020).

**Figure 3:**
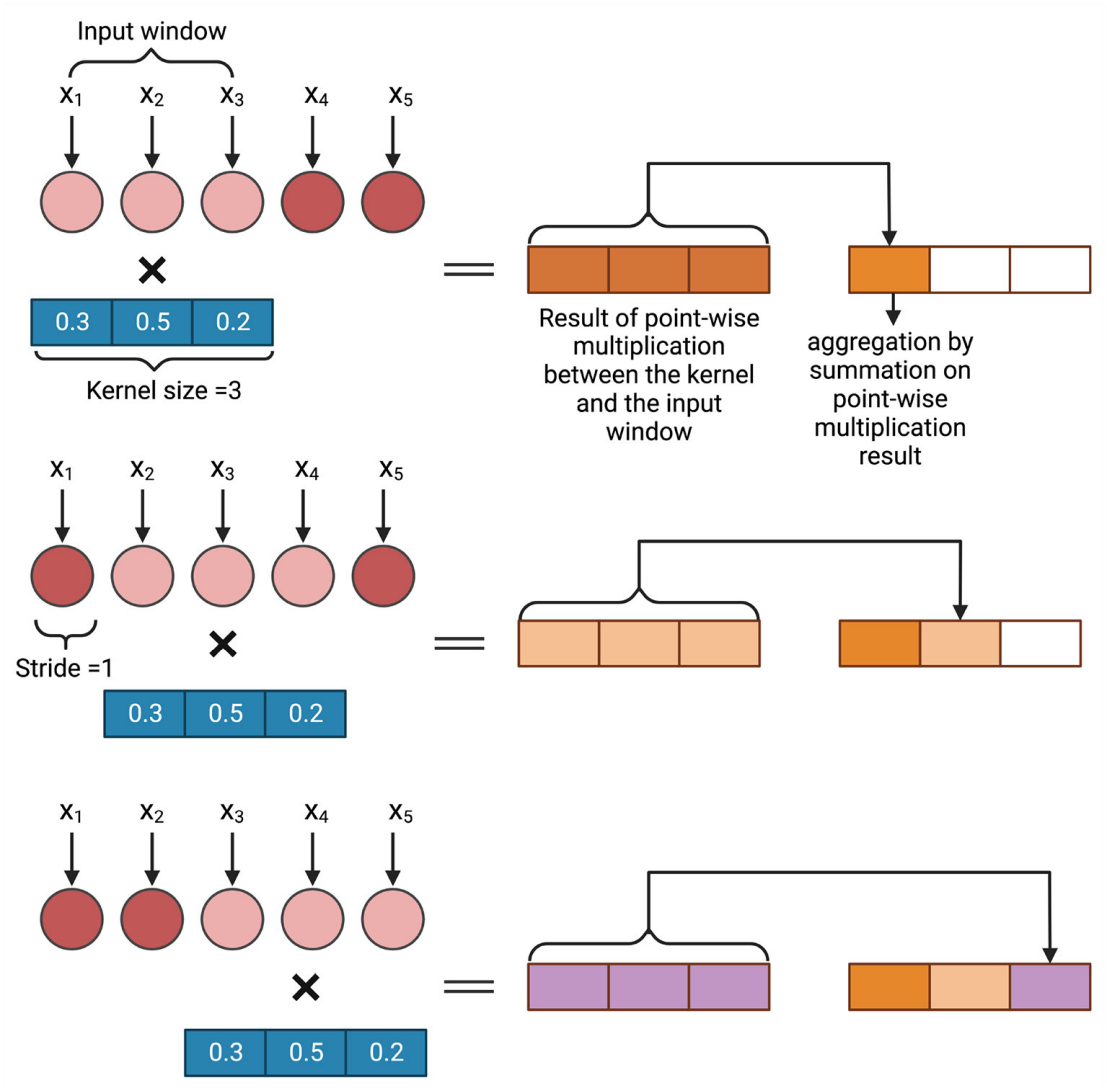
An example of convolution operation on a one dimensional input vector. In GS, *x*_*i*_ is a marker.

There are three types of convolution, conv1D, conv2D, and conv3D, available in different deep learning frameworks (Abadi, 2016; Paszke et al., 2017; Chollet, 2018). The choice of the convolution layer depends on the dimension of the input to the convolution layer. In GS, as the data is generally one-dimensional, conv1D is typically used (Ma et al., 2018). As the genotyped data is often categorical (1, 0, and –1), the marker data can also be converted to a one-hot encoded vector which will be the input of a conv2D layer (Liu et al., 2019b; Washburn et al., 2019; Avsec et al., 2021; Ji et al., 2021). Figure 3 shows an example of how 1D convolution works. In this example, a sequence of length 5 is processed with a kernel of size 3 and stride 1. The weights of the kernel are randomly initialized. A point-wise multiplication operation between the input window (in this example, the input window = 3) and the kernel takes place and after that an aggregation operation is performed. As the stride = 1, the input window then shift one space and the same operation of point-wise multiplication and aggregation takes place. This continues till the total input space is covered. The result is a sequence of length 3 where each neuron bears spatial information of the sequence.

To apply convolutional neural network for multi-environment trials, the algorithm should be developed carefully as a concatenated input vector of environment, genetic and soil data may not properly represent relationships between different data sources. The reason is that since the sliding window of convolution operation captures local information, a convolution operation on the concatenated vector may not properly reflect the effect of environment on the genetic data, as these are represented in regions of the concatenated vector that are not adjacent. To solve this problem, different types of neural networks can be employed on different types of data (Khaki et al., 2020; Washburn et al., 2021; Sharma et al., 2022). The predictions from different networks can be combined to obtain an overall prediction.

#### 3.1.3. Recurrent neural networks

Recurrent neural networks (RNNs) are distinct from both MLPs and CNNs as they are not feed-forward. Neurons in RNNs may have connections to themselves. RNNs are a family of neural networks, such as Long Short Term Memory (LSTM) (Hochreiter and Schmidhuber, 1997) and Gated Recurrent Unit (GRU) (Cho et al., 2014), that typically work with time-series and sequence data (Hochreiter and Schmidhuber, 1997). These networks have been successfully applied in weather prediction (Qing and Niu, 2018; Salman et al., 2018; Yu et al., 2019a) and in GS (Shook et al., 2020). Particularly, LSTM has been applied in genomic selection task mostly with environmental information (Shook et al., 2020). LSTM either preserves or forgets past information for future prediction by applying a particular structure called gates. The input of LSTM is time-steps or sequences and the output depends on all the previous time-steps or sequences. As LSTM are applicable to time-series data, the use with environmental data in GS allows the networks to efficiently summarize large-scale data. We refer the readers to the review on LSTM by Yu et al. (2019b) to know more about LSTM.

Generally, in multi-environment GS, historical weather information is the input to the RNNs. Genetic information is incorporated in the later part of the network (Shook et al., 2020). As the genetic information is not a time series data in nature, this part of the network generally does not contain any LSTM layers. The outcome is the predicted phenotypes for a specific weather condition.

#### 3.1.4. Transformers

Transformers are another type of neural networks that transform one sequence to another sequence. That is, the transformer architecture is designed to take a sequence as input but also produce a sequence as output (Vaswani et al., 2017; Ji et al., 2021; Jubair et al., 2021; Le et al., 2022), as opposed to a single value, which is the output of MLPs or and CNNs. The transformer architecture contains an encoder and a decoder. This encoder and decoder can be used separately or together. The transformer encoder has been applied in GS (Jubair et al., 2021) and other fields such as DNA representation learning (Ji et al., 2021; Le et al., 2022) and gene expression prediction of humans (Avsec et al., 2021). Here, we discuss only the transformer encoder to predict crop traits.

**Figure 4:**
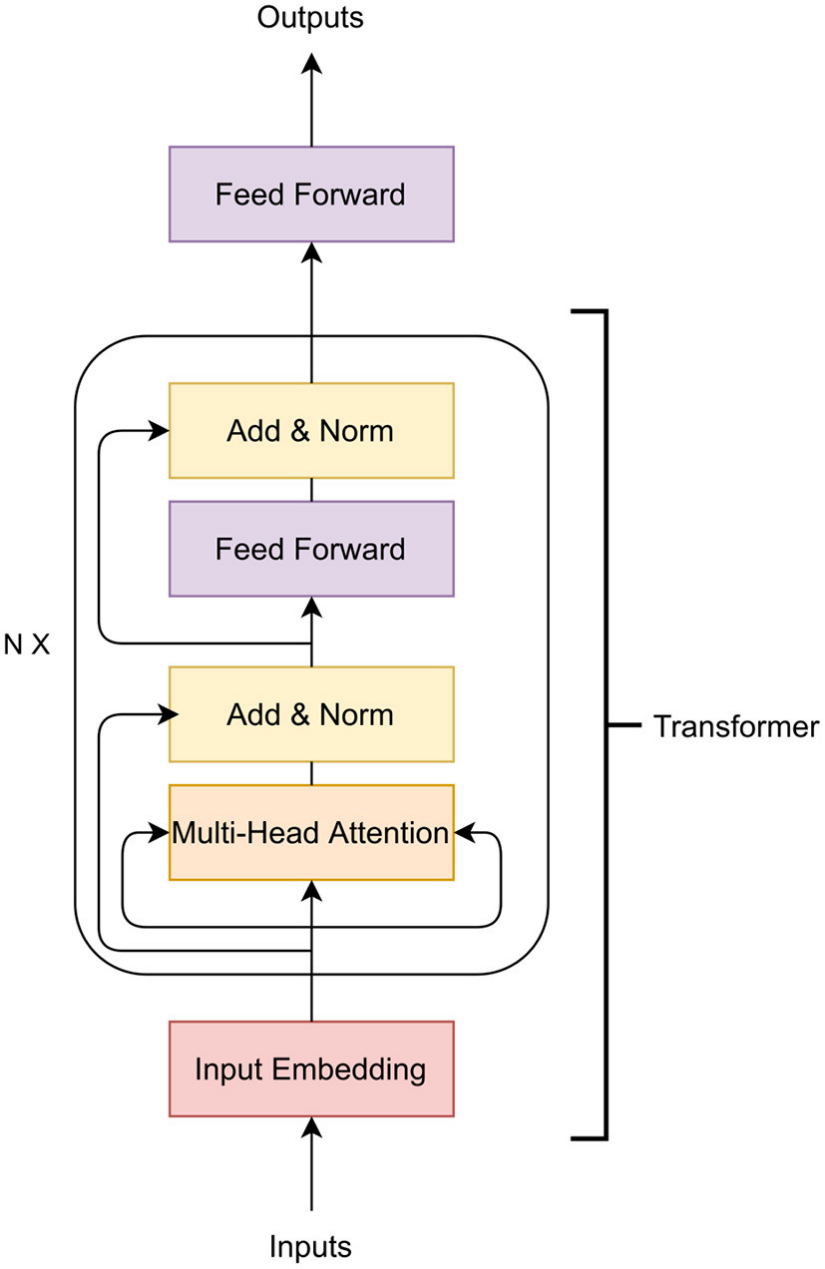
A transformer encoder. Here *N* represents *N* transformers can be stacked together.

The main building block of a transformer encoder is the multi-head attention layer which applies self attention (Vaswani et al., 2017). In GS, self-attention measures how important a marker is with respect to other markers for the phenotype prediction. Thus, the self attention captures the relationship of distant markers that influence the final phenotypic outcome (Jubair et al., 2021). Usually, the importance of markers with respect to a specific marker *m* is represented in a vector called attention vector. If multiple attention vectors are generated per marker, the final attention vector is the weighted average of all the attention vectors. The multiple attention vector is called multi-head attention. Apart from the multi-head attention layer, a transformer also contains a feed-forward neural network and layer normalization. Figure 4 shows a transformer encoder. The input of the transformer can be a one hot encoded vector or the genotype frequency (Avsec et al., 2021; Jubair et al., 2021). The embedding layer then embeds each marker to a *d* dimensional expanded representation. Usually a feed-forward neural network or a convolutional neural network is applied to embed the input features. The embedded representation of the markers are the input of the attention layers of the transformer.

### 3.2. Activation functions

The previous discussion shows that neural networks typically compute a linear function. However, as it is known that complex traits such as yields are non-linear, we need to introduce non-linearity in the network. Activation functions introduce non-linearity to the network by deciding which neuron should be activated. Each activation function addresses different limitations; see the survey of Szandała (2021) for information on different activation functions used in the literature. However, sigmoid, ReLU and tanh are the most widely used activation functions for GS (McDowell, 2016; Ma et al., 2018; Jubair and Domaratzki, 2019; Khaki and Wang, 2019; Khaki et al., 2020; Shook et al., 2020; Måløy et al., 2021; Washburn et al., 2021). Hence, we provide an overview of these activation functions below.

The sigmoid activation produces the output neuron between 0 and 1 by applying the sigmoid function (Szandała, 2021; Dubey et al., 2022). Though sigmoid function is one of the most used activation function, it suffers from the vanishing gradient problem, that is, the gradient of the loss function approaches zero, which causes the model parameters of the DNN to not update or update very slowly. It is also not zero centered, causing difficulties during optimization.

The tanh activation function solves the zero centered problem as the output of this function ranges from –1 to 1 (Szandała, 2021; Dubey et al., 2022). However, it suffers from vanishing gradient problem, as very high value and very low value of the input neuron will be mapped to –1 and 1 and other values will be toward zero.

ReLU (Rectified Linear Unit) is the most popular activation function which ranges from 0 to ∞ (Szandała, 2021; Dubey et al., 2022). It solves the vanishing gradient problem and because of the simplicity of the function, it converges quicker than other activation functions.

### 3.3. Regularization layer

A regularization layer helps DL algorithms avoid overfitting and leads to better generalization by reducing the model complexity (Kukačka et al., 2017; Moradi et al., 2020). The most popular generalization techniques employed in GS are L1, L2 and dropout regularizer. L1 regularization calculates the summation of the absolute value of the weight vectors while trying to estimate the median of the data. On the other hand, L2 regularization calculates the summation of the square of the weight vector that tries to estimate the mean of the data. Dropout (Srivastava et al., 2014) is the most popular regularization technique. Dropout regularization randomly drops a neuron with a probability *p* and thus reduces the complexity of the model.

### 3.4. Loss functions

A loss function calculates the loss between the observed phenotype and predicted phenotype during training. The most popular loss function for GS is mean squared error (MSE). MSE measures the average squared difference between the observed and predicted phenotypes (Rachmatia et al., 2017; Ma et al., 2018; Khaki and Wang, 2019; Khaki et al., 2020; Shook et al., 2020; Jubair et al., 2021). Categorical cross entropy has also been applied as the loss function where the prediction task is converted to a classification problem (González-Camacho et al., 2016).

### 3.5. Optimization

The objective of training is to optimize the DNN. For optimizing, after each iteration, the weights need to be adjusted to minimize loss function. An iteration over the whole training set is called an epoch. Optimizers adjust the weights by applying certain algorithms and optimizing the loss function (LeCun et al., 2015; Dong et al., 2021). Optimization functions typically apply gradient descent to optimize the weights of the neural networks. The gradient measured is in relation to the loss function, that is, between the true and predicted value of the network as it currently predicts at this point in training. Stochastic Gradient Descent (SGD) (Ruder, 2016) is an optimizer that uses a subset of the training data to calculate and update the gradient of each weight. It uses a hyper-parameter called the learning rate to control how much it will adjust the weights from each iteration. There are also some algorithms that employ an adaptive learning rate strategy such as Adagrad (Ruder, 2016) and Adam (Kingma and Ba, 2014). Instead of using a fixed learning rate for all the weights, they use different learning rates for each of them. Adam calculates the first and second moments of the gradients and updates weights based on this calculation. For more detail on Adam and other optimization methods, we refer the readers to the review by Sun (2020).

### 3.6. Performance metrics

Performance metrics measure the performance of a machine learning model on a test dataset, which indicates how well the model will perform in production. As the ultimate goal is to rank genotypes to create a new variety, most of the research applied a correlation based performance metric such as Pearson Correlation Coefficient (PCC), or a ranking based measure such as Normalized Distributed Cumulative Gain (nDCG) (Järvelin and Kekäläinen, 2017). Some research also applied MSE as the performance metric.

PCC measures how linear the predicted phenotypes and the true phenotypes. PCC values range from –1 to 1 where a perfect linear relationship is indicated by 1 and completely non-linear relationship is indicated by –1. The formula of PCC is given below:


$$r=\frac{\sum \left(x_{i}-\bar{x}\right)\left(y_{i}-\bar{y}\right)}{\sqrt{\sum {\left(x_{i}-\bar{x}\right)}^{2}\sum {\left(y_{i}-\bar{y}\right)}^{2}}}$$

In the above equation, *x*_*i*_ is the observed phenotype, $\bar{x}$ is the mean of observed phenotype, *y*_*i*_ is the predicted phenotype and $\bar{y}$ is the mean of predicted phenotype.

nDCG@k is a key measure for GS because it measures the quality of the ranking of the predicted phenotypes for the top *k* individuals (Järvelin and Kekäläinen, 2017; Jubair and Domaratzki, 2019). The formula for calculating *nDCG@k* is given below:


$$nDCG@k=\frac{DCG@k}{IDCG@k}$$

In the above equation, *DCG@k* means the discounted cumulative gain for the top *k* individuals. *DCG@k* measures the graded relevance of top *k* predicted genotypes. On the other hand, *IDCG@k* is the ideal *DCG* for the top *k* genotypes. The value of nDCG@k ranges from 0 to 1 where nDCG@k is 1 for perfectly ranked genotypes. nDCG was previously employed for measuring performance in GS by Ma et al. (2018) and then adopted by Jubair and Domaratzki (2019).

### 3.7. Training, test, and validation set

Supervised machine learning algorithms learn from the training data and their corresponding labels. Validation data is used to optimize the parameter of a machine learning algorithm while the final performance is measured on the test data. During training, the input of the DL algorithm is both genotyped and phenotyped data, with phenotypes being our target value to predict. An iteration for training a DL algorithm is called an epoch. After each epoch, the DL algorithm is validated on validation data to decide on the necessity of further training. During the validation step, the input to the DL algorithm is genotyped data while the model predicts the phenotypes. A loss between actual and predicted phenotypes for the validation data is measured. The training stops if there is no improvement in validation loss in *n* consecutive epochs. The final performance of the DL model is measured on the test data with the model that is obtained from the last most succesful epoch.

For a single environment trial, k-fold cross validation can be applied to divide the data into training test and validation sets. Runcie and Cheng (2019) recommended separating the training data and test data first and then applying k-fold cross validation on training data to divide the data in *k* training and validation sets (Refaeilzadeh et al., 2009).

For a multi-environment trial, a deep learning model can be evaluated in four scenarios, as described by Gillberg et al. (2019). In the first scenario, the authors used the trained model to observe the test lines in some environments. As some environments did not contain the test lines, the objective is to estimate traits of unobserved lines in those environments. In the second scenario, some lines are observed in some environments, but a subset of lines in the test set were never observed in any environments. The second scenario is more complex than the first one as the machine learning model has no prior knowledge of the test lines from any environment. In the third scenario, the machine learning model did not observe the environment where we want to grow the genotypes; however, the genotypes may be observed in other settings. The goal here is to predict traits for this new environment. Finally, the fourth scenario is the most extreme case of all scenarios. In this scenario, machine learning models do not have any prior information about the test lines and environment. That is, both lines and environments are new to the model and the objective is to predict traits for these new lines in a new environment.

In classical linear models, such as extensions to Genomic Best Linear Unbiased Prediction (GBLUP), environments are treated as a discrete category or as a relationship matrix between environments (de Los Campos et al., 2010; Endelman, 2011; Pérez and de Los Campos, 2014; Lopez-Cruz et al., 2015; Pérez-Elizalde et al., 2015; Crossa et al., 2016a; Cuevas et al., 2016; Hassen et al., 2018). Because of this, only the first two scenarios can be simulated, as environments unknown to the training set cannot be modeled. This demonstrates power of using deep learning models that are capable of incorporating heterogenous weather data directly into predictive models. In the examples we see in Section 5, deep learning models that incorporate weather data directly are capable of being evaluated in all four scenarios. However, the extent to which all these evaluations are performed varies.

## 4. Deep learning methods for single environment trials

**Figure 5:**
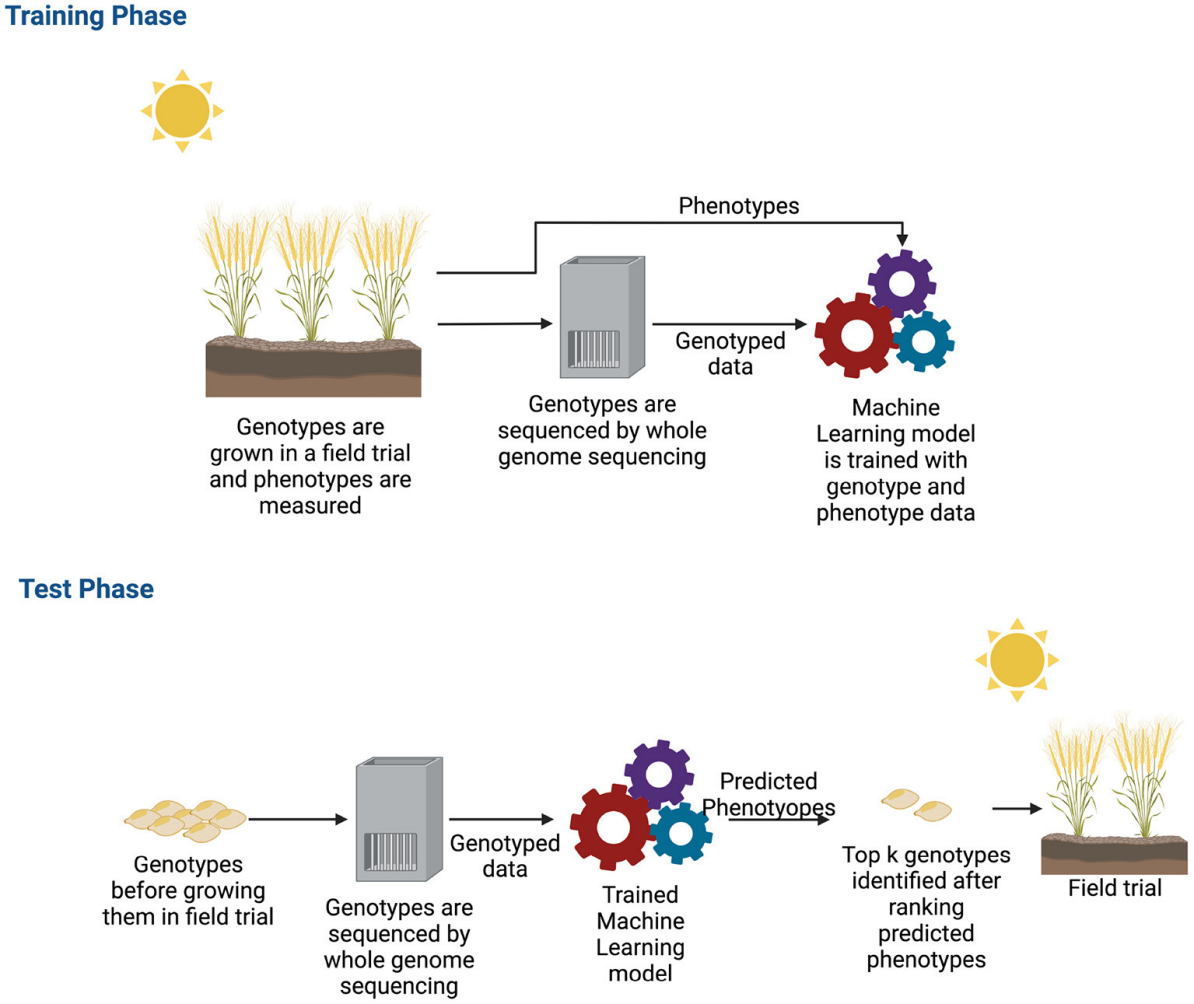
Workflow of genomic selection in single environment trial. For collecting training data, plants are grown in a field trial and phenotypes were measured. These plants are also genotyped. After obtaining both genotyped and phenotyped data, a machine learning model is trained during the training phase with both types of data. After the machine learning model is trained, potential genotypes that will be grown in the field are genotyped. These genotyped data are the input to the trained machine learning model. The machine learning model estimates the phenotypes. The estimated phenotypes are ranked and the top k phenotypes are chosen to select new varieties that will be grown in the field.

Single environment trials have been the subject of many approaches. The main objective of GS for a single environment trial is to build a new variety of crops for that specific environment. A variety of deep learning models have been demonstrated to be successful for single environment datasets and building a new variety for crops (Pérez-Enciso and Zingaretti, 2019; Tong and Nikoloski, 2021). During the training phase of a deep learning algorithm, the typical inputs to the neural networks are the genotyped data and phenotypes. The model learns from these observed data, and then, after learning, it predicts the phenotypes of unobserved genotypes. From the predicted phenotype values, the top *k* genotypes are chosen as potential candidates for new varieties. Figure 5 shows how a new variety is developed by applying machine learning.

DL models have received a significant amount of attention recently (Pérez-Enciso and Zingaretti, 2019) and can predict complex traits. DL methods have been mostly either based on fully connected networks or convolutional neural networks, with the exception of the early neural networks for genomic selection (Gianola et al., 2011; González-Camacho et al., 2012; Pérez-Rodŕıguez et al., 2012). Below, we discuss the advancement and motivation of different neural networks for single environment trials.

Early implementations of neural networks in GS were mostly based on Bayesian Regularization, known as Bayesian Regularization Neural Network (BRNN) and Radial Basis Function Neural Network (RBFNN). Since some phenotypes follow a Gaussian distribution for some species, this works as the motivation to apply BRNN and RBFNN. Bayesian Regularization assumes the weights of the neural network come from a Gaussian distribution and calculates the loss between predicted phenotypes and true phenotypes by applying the Bayesian probabilistic approach. RBFNN, on the other hand, applies the radial basis function on each hidden neuron and thus, works as an activation function. These networks usually have one input layer, one hidden layer and an output layer. Gianola et al. (2011) proposed a BRNN for genomic selection and applied their framework to predict wheat yield. They compared the model with Bayesian Ridge Regression and showed that 11–18% improvements with their BRNN depending on the number of hidden neurons. Pérez-Rodŕıguez et al. (2012) compared two different shallow neural networks: BRNN and RBFNN with linear statistical models such as Bayesian ridge regression (BRR) (Bishop and Tipping, 2003), Bayesian LASSO (Hans, 2009), BayesA (Meuwissen et al., 2001), and BayesB (Meuwissen et al., 2001) on twelve different single environment trials and two phenotypes, grain yield and days to heading. Though there was no single winner for all traits and phenotypes, the research showed that non-linear models perform better than linear statistical models in general. Similar research is conducted by González-Camacho et al. (2012), which applied RBFNN on twenty-one traits of maize. The results showed that RBFNN performs similarly or better than statistical models.

After the moderate success of BRNN and RBFNN, researchers have applied shallow fully connected neural networks to GS. The shallow fully connected neural networks usually contain one or two hidden layers. González-Camacho et al. (2016) conducted a large study between a probabilistic ANN (PNN) and shallow MLP model on 33 datasets comprising wheat and maize. The PNN model is the extension of RBFNN where a softmax activation function is applied to convert the output of the RBF kernel layer to a probability of *c* classes. The shallow MLP model consists of two hidden layers and also predicts a class as the output. As their model predicts a class, they transformed the regression problem into a classification problem by dividing the data into three categories, where the top category contains 30%, the middle category is 40% and the bottom category is the remaining 30%. The results showed that the PNN is better than the shallow MLP model for classification.

McDowell (2016)'s M.Sc. thesis also employed three shallow fully connected neural networks to GS consisting of one to three hidden layers. In their shallow models, they also employed different regularization techniques such as L2 and dropout regularization on some benchmark datasets, such as wheat and maize. Overall, the single hidden layer regularized neural networks performed better than the unregularized ones. The research showed that though increasing the number of hidden layers decreases the performance of their model, the neural networks are as good as the statistical models.

Rachmatia et al. (2017) proposed a different model than MLP known as Deep Belief Network (DBN). The motivation of applying DBN is to learn the genetic structure of the genomic data for a specific phenotype prediction. DBNs are usually applied in a semi-supervised setting where only a limited portion of the data is labeled. Thus, from all the available genomic data, it first tries to identify the pattern within the data by applying Restricted Boltzman Machine (RBM) (Zhang et al., 2018) blocks. Each RBM block in the DBN focuses on learning the probability distribution of its previous layer and, in the end, produces a feature vector for each input. This feature vector is the input to an output layer that predicts the phenotypes. Rachmatia et al. (2017) employed three block RBMs to predict both additive and non-additive effect phenotypes of maize, such as grain yield, female flowering, male flowering, and the anthesis-silking interval. The results showed that while the DBN is better than the existing statistical methods (BLUP and Bayesian LASSO) for predicting non-additive phenotypes, the performance for additive phenotypes drops significantly below BLUP by 3.5–7.5% for different traits.

Though most of the research has found that machine learning performs better than the statistical methods (McDowell, 2016; Rachmatia et al., 2017; Ma et al., 2018; Montesinos-López et al., 2019a) found that statistical methods are as good as machine learning methods and that SVMs (Hearst et al., 1998) are better than fully connected deep learning models. However, they also discussed the reason for the low performance of DL methods might be because of the small dataset they used, which only contained 270 wheat lines.

To the best of our knowledge, DeepGS (Ma et al., 2018) was the first method that applied CNN for GS. As GS data are high dimensional, DeepGS employed a combination of convolution, dropout and pooling layers. Conceptually, the adoption of CNN, with strides and window size, allows the possibility to integrate the effect of proximal markers and later when a linear layer is applied, capture the overall influence of markers on the phenotype. Ma et al. (2018) used a ranking procedure called Mean Normalized Cumulative Gain to rank the predicted individuals and obtained 2–7% improvements in the ranking of traits such as grain length, grain width, grain hardness, thousand-kernel weight, test weight, sodium dodecyl sulfate sedimentation, grain protein, and plant height, compared to RR-BLUP. They also showed that the selection of input markers and reducing the data dimension improved the performance of the deep learning model.

Jubair and Domaratzki (2019) proposed an ensemble CNN model to predict six traits of wheat. Each CNN model in the ensemble is created by a subset of randomly selected markers from the marker set. The final output is the average of the models in the ensemble. They compared their model with other non-ensemble and ensemble machine learning methods such as: support vector regression (SVR), CNN, ensemble SVR and Random Forests (Breiman, 2001) and RRBLUP. The work showed that overall ensemble machine learning methods are 20–30% better than single machine learning methods and slightly better than RRBLUP in correlation coeffcient and genotype ranking. The notable observation from this research is when CNNs are applied on a random marker set, the model still performs well, indicating little importance of the spatial relationship of GS for wheat. This observation also aligns with the observation of Ma et al. (2018).

Liu et al. (2019b) applied a dual-CNN architecture where after the input layer, they applied two separate streams of CNN that are not connected. The first stream has two CNN blocks and the second stream has one CNN block. The motivation behind employing two CNN streams is to use the second stream as a residual connection to the first CNN stream by aggregating two CNN streams together. The aggregated output is then passed to another CNN block, followed by a fully connected block for further processing and predicting phenotypes. Their model is trained and tested on a soybean dataset which performs better than DeepGS (Ma et al., 2018), MLP and statistical methods such as RRBLUP, BRR, BayesA, and Bayesian Lasso. The saliency map they applied also showed that the dual stream CNN model puts more importance on known biologically important markers for the specific traits.

There have been some other researches that employed CNN with limited success. Zingaretti et al. (2020) applied CNN in two polyploid species: strawberries and blueberries for predicting five different phenotypes. Their study showed that while CNN outperformed statistical models and Reproducing Kernel Hilbert Spaces (RKHS) for epistatic traits, it was not as successful for additive and mixed traits. Pook et al. (2020) showed the importance of dataset size while applying CNN in genomic selection. In an arabidopsis dataset, they showed that increasing training data could allow a CNN model to outperform state-of-the-art models such as GBLUP and MLP. Sandhu et al. (2021c) applied MLP and CNN on multiple traits of spring wheat data. Their research showed that no unique MLP or CNN models worked well with all traits, since the number of hidden neurons, activation functions and the number of hidden layers differs from trait to trait. While there is 0 to 5% improvement in correlation score from RRBLUP with CNN and MLP, MLP performs consistently better than CNN by a very small margin.

Self-attention is a recent mechanism in DL which identifies the relationship among features and has been applied primarily to natural language processing (Devlin et al., 2018; Liu et al., 2019a; Raffel et al., 2020). One of the popular methods for incorporating self-attention is the transformer model. Though the transformer and attention have not been the subject of much research for GS, they have been applied successfully in similar research areas (Avsec et al., 2021; Ji et al., 2021; Le et al., 2022). Jubair et al. (2021) proposed a transformer-based DL method for genomic selection. The main motivation for employing the transformer in genomic selection was to capture and use the information on internal relationships between markers to predict phenotypes. To the best of our knowledge, this was the first transformer-based DL method for GS in a single environment trial. The model was trained on a barley dataset to predict Fusarium Head Blight (FHB) and Deoxynivalenol (DON) content in barley. Their work showed that even with a small amount of data (400 genotypes), the transformer-based DL method can be as good as or better than the state-of-the-art GS methods such as BLUP. It also outperformed other machine learning methods such as MLP, linear regression and decision trees. However, the authors also mentioned the limitation of the transformer in terms of memory and time complexity, as it needs a massive amount of memory and computation time and may not be feasible to consider all markers representing the whole genome.

Montesinos-Lopez et al. (2021) proposed an MLP model that applied negative log-likelihood of Poisson distribution as the loss function to predict counts of symptomatic spikelets of Fusarium Head Blight (FHB) in wheat in three different environments. The model was compared with the MLP model without the Poisson loss, Generalized Poisson Ridge regression, Generalized Poisson Lasso regression, Generalized Poisson Elastic net regression, Bayesian normal Ridge regression and Bayesian log normal Ridge regression. The MLP model with negative log-likelihood of Poisson distribution loss was better than the normal MLP model and performed similarly to Bayesian normal Ridge regression. The use of Poisson distributions in this research was motivated by the particular phenotype of FHB-affected spikelets: Poisson distributions are an accurate model for situations when counting of some quantity. The authors note that this extends beyond physical counts (as of spikelets) but to other situations as well, like laboratory test results and adverse drug events. Further attention is necessary for integrating Poisson models, as they are not commonly used in many datasets that fall into these categories.

Ubbens et al. (2021) also explained deep learning for GS. The work examined a kernel method for masking marker data while making prediction, to investigate the role that other factors, such as marker location, play on prediction. The authors concluded that deep learning models for GS may suffer from so-called shortcut-learning (Geirhos et al., 2020), where models learn from contextual information that is correlated with the outcome variable rather than the intended data, which in this case is the marker data. This suggests that further attention is necessary for using deep learning with GS. This also gives motivation for incorporating environmental data into models, as this yields larger data set and may mitigate overfitting.

## 5. Deep learning methods for multi-environment trials

**Figure 6:**
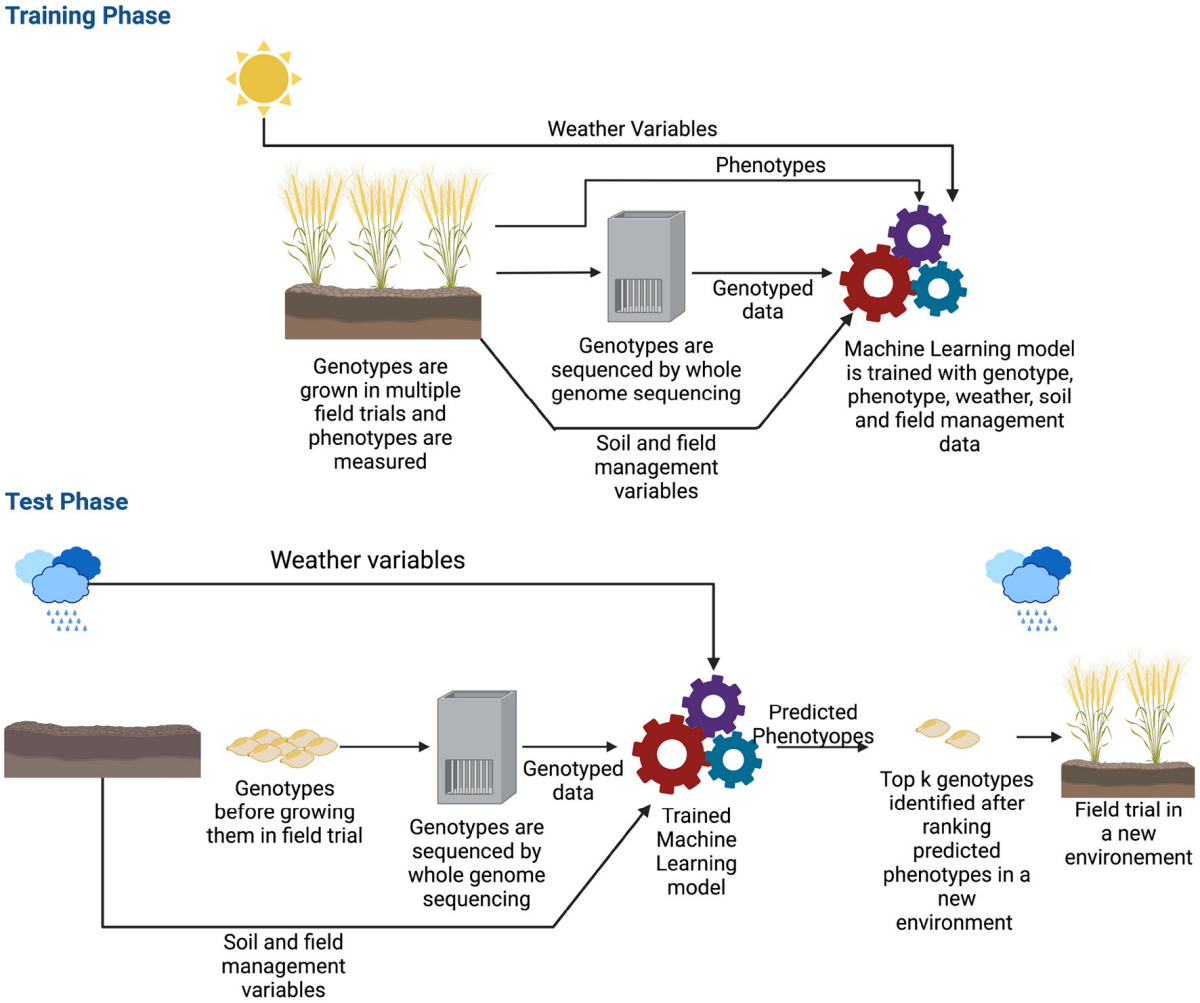
Workflow of genomic selection in a multi-environment trial. Before training the machine learning model, along with genotyped and phenotyped data, environmental information such as weather variables, soil and field management data are also collected. The genotypes are typically grown in multiple seasons/locations which provides a wide range of environmental data. During the training phase, the model is trained with all these data along with phenotypes. After the model is trained, in production, the model is given the genotyped data of crops along with environmental information of where the crops will be grown as the input. The model estimates the phenotype for that environment. Based on the estimated phenotypes, the top k genotypes are chosen and grown in the field.

The previous section shows that deep learning methods can predict complex traits in a single environment trial. However, extending models to multi-environmental datasets is challenging (Oakey et al., 2016; Crossa et al., 2017; Rincent et al., 2017). Here, a multi-environment deep learning model is defined as a deep learning architecture that takes environmental and/or genetic data as the input and predicts phenotype for a specific environment. Though the ideal scenario is training a model with genotyped data along with weather, soil and field management information (Khaki and Wang, 2019; Washburn et al., 2021), some of this data is sometimes not available and some of the multi-environment models are developed with environmental data only (Khaki et al., 2020; Lin et al., 2020; Shook et al., 2020; Zhong et al., 2022). Since in a multi-environment task, our goal is to estimate phenotypes of a crop in a new environment, the machine learning model typically needs field trialed data in many different environments. An environment is the growing cycle of a crop; for example, if a crop is grown multiple times of the year in the same field, each instance will be a different environment. As crops need to be grown numerous times in various locations, collecting these data may take years before it is possible to train a machine learning model (Spindel and McCouch, 2016). In addition, as the sources and types of data are different (genetic, weather, soil and field management data), the machine learning model can become very complex. Figure 6 shows the workflow of a multi-environment trial.

We have discussed single trait trials, where the deep learning model estimated one phenotype. There have been studies that develop multi-trait deep learning models for multi-environment trials, to predict multiple phenotypes simultaneously. The intuition behind this approach is that deep learning models will capture the information of common factors as well as phenotype-specific factors to predict phenotypes. Montesinos-López et al. (2019c) proposed an MLP containing three hidden layers and an output layer with three neurons to predict grain yield, days to heading and plant type of wheat. The input to this model is the concatenated matrix of environmental variables, a genomic relationship matrix obtained from genotypes, and a GxE term. The model was compared with GBLUP and MLP for the single phenotype. They observed that multi-trait MLP is better than the single trait MLP and overall, GBLUP model outperformed all of them with limited data (259 lines). Guo et al. (2020) also applied the same architecture of a multi-trait MLP model with a minimal wheat dataset (240 genotypes). Though their dataset was different than Montesinos-López et al. (2019c), as it consisted of genotyped data and environmental information, they also observed better performance. Sandhu et al. (2021a) applied the same MLP architecture on a wheat dataset comprised of spectral information of site-year and genetic information. These data were concatenated together to predict yield and protein content. The notable difference between this work and the previous two (Montesinos-López et al., 2019c; Guo et al., 2020) is the amount of data, as their dataset comprises 650 genotypes. The work showed that MLP performs similarly or better than GBLUP, BayesA, BayesB (Meuwissen et al., 2001), Random Forests (Breiman, 2001), CNN and Support Vector Machines (Hearst et al., 1998).

The model of Khaki and Wang (2019) was the first research to incorporate genetic information of corn and rich weather and soil data into a single deep learning framework. Their proposed method has two disjoint parts: (i) predicting weather variables for the growing cycle and (ii) predicting yield. In the first part, they employed individual shallow MLP that take the previous 4 months' data of a specific weather variable as the input to predict the monthly weather variables of the growing cycle. In the second part, their deep learning model for predicting yield contained 21 fully connected neural network blocks where each block had 50 hidden neurons, an activation function and a regularization function. The input of this network was a concatenation of genetic information and weather variables obtained from the first part, along with soil information. The predicted output was the yield. As each hidden neuron combined environmental and genetic information, the motivation was to capture the GxE in each hidden neuron to predict yield. This model improved the correlation coefficient between predicted yield and original yield by 57% compared to the model that only had genomic data as the input.

Shook et al. (2020) proposed an LSTM-Fully Connected Neural Network based deep neural network that processed the inputs in two stages to predict soybean yield. In the first stage, LSTM blocks were employed on historical weather data. The weather data was divided into multiple time steps in the growing season where each time step is 30 days. An average of each weather variable was taken within the given time steps. LSTM blocks were applied on all the times steps to capture the temporal relationship and provide a context vector as an output optimized for yield prediction. After obtaining the context vector, maturity group information and a genotype cluster derived from applying k-means on the pedigree matrix were concatenated with the context vector. This concatenated vector was the input of the fully connected network that predicted yield. This model showed that when cluster and maturity group information are added, it leads to a lower root mean square error (RMSE).

Deep learning has also been successfully applied when no genetic or pedigree information is available. The deep learning model of Lin et al. (2020) had two parts: (i) attention-based LSTM network that captured the effect of environmental variables over time on yield, and (ii) multi-task learning (MTL) networks that predicted location-specific corn yield anomaly. The weather information was the weekly average of minimum and maximum temperature, precipitation, growing degree days and killing degree days. This model was compared to Random Forests and Lasso (Ranstam and Cook, 2018) and had the lowest RMSE among the three.

Khaki et al. (2020) employed a CNN-RNN based deep learning model on a dataset that contained historical yield and weather information and soil data for corn and soybean. In this work, CNNs were applied to yearly data to capture the spatial information of weather and soil information. Two separate CNN networks were employed that output two vectors to capture the spatial information of weather and soil variables. After obtaining the spatial information, LSTMs were applied to obtain the temporal relationship within the data. To employ LSTM, the distributed representations of soil and weather along with the corresponding yield of previous *t* years were concatenated and provided as the input to the LSTM, which predicted the yield of the current growing cycle. This model improved the correlation coefficient by 20–25% compared to LASSO (Ranstam and Cook, 2018) based on different years and crops.

Gangopadhyay et al. (2020) applied a dual attention neural network on a soybean dataset that comprised 13 years of data of 5,839 genotypes resulting in 103,365 observations. The attention networks are known for their ability to identify important features as it calculates an importance score (attention score) for each feature and aggregate all the features in a context vector by applying weighting based on the attention score. The dataset contained weekly weather variables such as average direct normal irradiance, average precipitation, average relative humidity, maximum direct normal irradiance, maximum surface temperature, minimum surface temperature and average surface temperature. A fully connected neural network followed by an attention layer was applied initially to the weather variables to capture the spatial information. Then, on the output of the spatial attention layer, multiple LSTM layers followed by another attention layer were applied to capture the temporal relation and predict the soybean yield. Though their model had comparable performance to the baseline model (LSTMs and LSTMs with temporal attention), they showed that the attention layer provided their model with more interpretability. They also observed that the attention mechanism identified average precipitation as the most influencing factor for soybean growth in most weeks.

McCormick et al. (2021) applied nine different architectures of LSTMs to predict the current growth stage of soybean. The architectures of LSTMs mostly differ in the number of layers and hidden neurons. These models were applied to a dataset consisting of 187 environments and 13,673 observations of soybean, based on different planting times and locations. Their weather variables included daily minimum and maximum temperature, solar radiation, night length, longitude and latitude. The task of these LSTM models was to predict, from seven growth stage variables, what the current stage of the plant is. In their LSTM model, they also included the output of a knowledge-based model named CROPGRO (Boote et al., 1998; Salmerón and Purcell, 2016) as features and showed that including the predicted output from CROPGRO as a feature improved the mean absolute error by 2.76 and 5.51% for different traits.

Washburn et al. (2021) applied a CNN-MLP based neural network on maize data. Their dataset is similar to Khaki and Wang (2019) as their data contains genetic, environmental, soil and field management information. Initially, this model processed the inputs in three parts: (i) fully connected blocks were applied to genetic data, (ii) CNN blocks were applied to environmental information and (iii) fully connected neural network blocks were employed on soil and field management data. Then the outputs of these three parts were concatenated and passed to fully connected blocks to predict yields. They observed that soil and environmental factors play a bigger role than the genetic information for yield prediction as they comprised 35 and 22% of the importance score, respectively. From the feature perspective, precipitation, vapor pressure and plant density were the most influential features. They also observed that adding historical information for a specific location improved prediction and overall, the performance of the proposed CNN-MLP model was comparable to or better than GBLUP-based models.

Måløy et al. (2021) employed a variation of transformers named performers (Choromanski et al., 2020) on a barley dataset to predict yield. Performers were developed as attention-based models capable of capturing long-range interactions between features; this is appropriate for genomic data where attention related SNPs may be distant in the genome. In their work, the environment variables were of two types: (i) mean value of temperature and precipitation for the entire growing season and (ii) mean temperature and cumulative precipitation for each day of the growing season (historical data). Performers were applied to the genomic data to extract genomic features. An MLP was employed when the mean weather variables for the entire growing season were considered, or a performer was employed when historical weather data was considered as the input, to obtain the relevant features from the weather variables. Finally, both feature representations were concatenated and passed as the input to the regression layer to predict yield. Their results demonstrated that the model that considered historical weather information had the highest *R*^2^ scores. Their model also outperformed a CNN + MLP model by 1.3% in *R*^2^ score. In addition, as the historical weather data based model was better than average weather based models, the results showed that research needs to concentrate on integrating historical weather data and genomic data together in a meaningful way for different growth stages of crops to predict genotype-specific yield for a specific environment.

Zhong et al. (2022) proposed a multi-task learning model where each task-specific layer predicted the average yield of maize for a specific county. Their input variables contained weather, remote sensing and soil data. K-means clustering was applied to county-level yield and weather and soil data to obtain spatial features. In addition, an LSTM and a fully connected neural network were applied to the weather data and soil data, respectively, to extract temporal and soil features. Finally, these three outputs were combined and served as the input to the county-specific output layer that predicted yield for that specific county. The result of the proposed model showed that killing degree days was one of the major driving factors for yield loss in 2012. As this model predicted county-specific yield, it did not integrate genetic information. However, this model considered spatial-temporal relationships which can be integrated with genomic data and have the potential to play a vital role in capturing GxE.

Sharma et al. (2022) proposed a deep learning model that contains four modules: genome, weather, field management and soil module and predicted maize yield. For each of these modules, they obtained an embedded vector representing the feature set of that module by employing different types of neural networks. For example, two different CNNs were employed for weather and genomic data, while two separate MLPs were used for field management and soil data to obtain embeddings for each module. In addition, they applied an attention mechanism between the genome embedding and weather data embedding to learn an embedding that replicates GxE. Finally, the embeddings for GxE, weather, field management and soil were concatenated, and a fully connected layer was employed to predict the yield. The results demonstrated 1.45 times better correlation coefficient than GBLUP and CNN-based methods. This approach is unique compared to other methods as they used the attention mechanism to obtain GxE, which ideally puts more importance on the environmental variables that influence maize yield.

In Table 3, we list the deep learning-based academic papers that work with multi-environment trial and environmental data. Some single-environment models (Sandhu et al., 2021b,c, 2022) employed an MLP, similar to the model of Montesinos-López et al. (2019c), to predict quantitative traits in another location or year. As these models did not incorporate environmental data into the model, we consider them single-environment models. Thus, this type of research, while important in demonstrating advances in prediction of traits in new situations, is not summarized in this survey. In addition, typically, environmental information is not readily available, and even if they are available, these models are complex in nature as different types of data need different types of ANNs to extract meaningful features. Thus, the development of new deep learning approaches in this new research area is comparatively slower than single environment trial models. We expect that, as data collection and integration continues in crop breeding programs, more detailed datasets containing rich genotypic, weather, soil and management data will be generally available. Models that incorporate this data will become more common as well, as the data becomes more reliable, standardized and available.

**Table 3:** Papers on multi-environment deep learning models.

**Year**	**References**	**DL model**	**Crops**	**Traits**	**Geno** **data**	**Weather** **data**	**Soil** **data**	**Other** **data**
2019	Khaki and Wang (2019)	MLP	Corn	Yield	Yes	Yes	Yes	
2019	Montesinos-López et al. (2019c)	MLP	Wheat	Yield, Days to Heading	Yes	Yes	No	
2020	Shook et al. (2020)	LSTM- MLP	Soybean	Yield	No	Yes	No	Genotype Cluster
2020	Khaki et al. (2020)	CNN- RNN	Corn, Soybean	Yield	No	Yes	Yes	Historical Yield, Field Management
2020	Lin et al. (2020)	Att- LSTM	Corn	Yield	No	Yes	No	
2020	Gangopadhyay et al. (2020)	MLP LSTM Att	Soybean	Yield	No	Yes	No	
2020	Guo et al. (2020)	MLP	Wheat	Yield, Harvest Index, Spike Fertility, Thousand Grain Weight	Yes	Yes	No	
2021	Sandhu et al. (2021a)	MLP	Wheat	Yield, Protein Content	Yes	Yes	No	
2021	Washburn et al. (2021)	CNN MLP	Maize	Yield	yes	Yes	Yes	Field Management
2021	Måløy et al. (2021)	Transformers MLP	Barley	Yield	yes	Yes	No	
2022	Zhong et al. (2022)	LSTM MLP	Maize	Yield	No	Yes	Yes	
2022	Sharma et al. (2022)	CNN MLP Att	Maize	Yield	Yes	Yes	Yes	Field Management

In the table, MLP means Fully Connected Networks and Att means attention networks.

## 6. Discussion

Genomic selection is a well-established tool for crop breeding, and non-linear supervised deep learning models are increasingly being used to predict phenotypes for complex traits. As datasets become increasingly feature-rich and large enough to train complex models, the use of deep learning models becomes more feasible. This trend also enables incorporating heterogeneous weather, soil and field management data to be added to predict environmental effects on genotypes. Typically, weather variables such as precipitation and vapor pressure (Gangopadhyay et al., 2020; Washburn et al., 2021) are the most important. However, other environmental variables such as day length (Tacarindua et al., 2013; Rahman et al., 2018; Islam et al., 2019), and maximum and minimum temperature (Gul et al., 2020; Moore et al., 2021) may also become vital based on the crop species and environment. These weather variables are the most influential during the early stages of crop development (Washburn et al., 2021). As these weather variables are mostly available as hourly or daily data, determining how this information can be added to the deep learning models, especially during the early stages of development, is essential (Gangopadhyay et al., 2020). Most existing methods employed neural networks on monthly average data of weather variables for the whole growing season (Khaki and Wang, 2019; Khaki et al., 2020; Shook et al., 2020). To add more information in the early stage of development, a variable length time window approach can be adopted where in the beginning, time window can be shorter, and in the later stage, the size of time window can be increased. Additionally, the use of unsupervised learning techniques to learn appropriate representations of weather data is a potential area of additional exploration.

Some research (Khaki and Wang, 2019; Washburn et al., 2021) incorporated a wide range of soil and field management variables in their model, such as soil electrical conductivity, calcium carbonate content, saturated hydraulic conductivity, gypsum content, plant density, irrigation, and pH. Typically, water and nutrition-related soil variables are the most relevant (Washburn et al., 2021). Though it is observed that soil variables are more important than weather variables (Washburn et al., 2021), in most of the current research, these variables are not considered due to the lack of data. Recently, the use of IoT devices to collect soil and field data (for example, weather variables described above) is gaining popularity (Sharma et al., 2020). As IoT devices can collect data more accurately and frequently, it has become possible to estimate soil nutrients and moisture for the growing cycle (Sharma et al., 2020). These estimated values can be the input of the deep learning algorithm to estimate phenotypes. Another source of data that can work as the input of GS is high-quality image data of fields. Drones with high-quality cameras have been used recently to capture field images. These images can be fed into a deep learning model to add additional information about the field. Recent research has indicated that using early phenotypic data, including spectral data collected by drones, yields models that can be competitive with GS (Adak et al., 2021) in predicting phenotype at harvest. Since GS aims to estimate yield even before sowing, we need to ensure that the information added in the model is collected either before sowing the plants or is estimated for the growing season based on previously available data. Collecting phenotypic information during growing season to attempt to predict future phenotypes represents a different philosophy of approaching GS, either when this data is used alone or in conjunction with genomic data. This approach may be consider advantageous in forestry or perennial crops, where early phenotypic information may shape long-term field trials (Cros et al., 2015; Kwong et al., 2017; Faville et al., 2018; Crain et al., 2020; Lebedev et al., 2020; Archambeau et al., 2022).

Most of the multi-environment deep learning architecture we discussed so far sought to capture the spatial and/or temporal effect of environmental variables on traits and later incorporated genomic data into the model for estimating phenotypes. Though a few deep learning models were developed by employing attention for genomic selection (Gangopadhyay et al., 2020; Jubair et al., 2021; Måløy et al., 2021), we believe attention-based architectures are the most promising approach for genomic selection. Attention-based methods can capture both temporal and spatial information and summarize the input data by aggregating them based on importance scores. As a robust model needs to be trained on different types and data sources, attention may play a significant role by providing more importance to the critical parts of different data sources (Gangopadhyay et al., 2020; Jubair et al., 2021; Måløy et al., 2021).

As one of the major challenges of GS for multi-environment is the data, collaboration among breeders and a well-defined data collection strategy will be useful to take GS application into production (Spindel and McCouch, 2016; Xu et al., 2022). To the best of our knowledge, the only user-friendly software designed to integrate multiple data sources in genomic selection is learnMet (Westhues et al., 2022). This software allows the user to employ traditional machine learning methods, such as XGBoost and Random Forests, and MLP-based neural networks. However, complex models also need to be packaged as user-friendly software to make more accurate predictions and bring GS to breeders.

In summary, continued advances in deep learning, driven by disparate application areas such as vision and languages, will continue to be adapted to GS, especially in the context of large datasets incorporating environmental conditions. Future research should focus on extracting meaningful features from different data sources and leveraging their interactions to predict quantitative traits. To extract meaningful features, choosing an appropriate deep learning architecture that can capture different relationships within each type of data will be the first step. For example, weather and image data during the growing season contains a spatial-temporal relationship, whereas soil data before the growing season has a spatial relationship. There are also heterogeneous unstructured text data about field management, such as the sowing pattern of crops, the amount of water supplied during irrigation, and notes on the overall condition of fields. Deep learning architecture such as transformers may play a vital role as they have been successfully employed to extract meaningful features from genomic (Avsec et al., 2021; Ji et al., 2021; Monteiro et al., 2022), weather (Måløy et al., 2021), and unstructured text data (Devlin et al., 2018; Raffel et al., 2020). However, GS for multi-environment model may need to employ different types of neural networks on different sources of data depending on the data property, such as the spatial, temporal and spatial-temporal relationship between variables. Future research also should focus on how to capture the interrelationship between genotypes and these features to predict quantitative traits.

## Author contributions

SJ: formal analysis, investigation, and writing—original draft. MD: writing—review, editing, and supervision. All authors contributed to the article and approved the submitted version.
